# Getting the Entire Message: Progress in Isoform Sequencing

**DOI:** 10.3389/fgene.2019.00709

**Published:** 2019-08-16

**Authors:** Simon A. Hardwick, Anoushka Joglekar, Paul Flicek, Adam Frankish, Hagen U. Tilgner

**Affiliations:** ^1^Brain and Mind Research Institute, Weill Cornell Medicine, NY, United States; ^2^Garvan Institute of Medical Research, Sydney, NSW, Australia; ^3^European Molecular Biology Laboratory, European Bioinformatics Institute, Hinxton, United Kingdom

**Keywords:** RNA, isoforms, long-read, splicing, epitranscriptome

## Abstract

The advent of second-generation sequencing and its application to RNA sequencing have revolutionized the field of genomics by allowing quantification of gene expression, as well as the definition of transcription start/end sites, exons, splice sites and RNA editing sites. However, due to the sequencing of fragments of cDNAs, these methods have not given a reliable picture of complete RNA isoforms. Third-generation sequencing has filled this gap and allows end-to-end sequencing of entire RNA/cDNA molecules. This approach to transcriptomics has been a “niche” technology for a couple of years but now is becoming mainstream with many different applications. Here, we review the background and progress made to date in this rapidly growing field. We start by reviewing the progressive realization that alternative splicing is omnipresent. We then focus on long-noncoding RNA isoforms and the distinct combination patterns of exons in noncoding and coding genes. We consider the implications of the recent technologies of direct RNA sequencing and single-cell isoform RNA sequencing. Finally, we discuss the parameters that define the success of long-read RNA sequencing experiments and strategies commonly used to make the most of such data.

## An Abundance of Alternative RNA Processing Events

The first decade of the new millennium has made it abundantly clear that most genes produce multiple distinct isoforms: Estimates of the fraction of multi-exon genes that are alternatively spliced rose from 42% in 2001 (Modrek et al., 2001) to 74% in 2003 (Johnson et al., 2003), to 86% in 2006 (Harrow et al., 2006). The new technology of RNA-sequencing (Mortazavi et al., 2008; Nagalakshmi et al., 2008; Pan et al., 2008; Sultan et al., 2008; Wang et al., 2008; Wilhelm et al., 2008) and its application to alternative splicing finally pushed this estimation to 95–98% (Pan et al., 2008) and 98–100% (Wang et al., 2008) in 2008. Simultaneously, the RNA community has established the existence of more than 2 million RNA editing sites (Ramaswami and Li, 2016), and that the number of transcription start sites (TSS) outnumbers by an order of magnitude the number of genes (Forrest et al., 2014), implying widespread alternative TSS usage. Also, polyA-site estimates are on the rise, with more than half of all genes now known to have alternative polyA-sites (Tian et al., 2007; Sandberg et al., 2008; Mayr, 2016). Taken together, these observations reveal a vast abundance of alternative processing events that can affect RNA molecules. Beyond the four-letter sequence of RNA molecules, chemical modifications on RNA nucleotides, collectively referred to as the “epitranscriptome” (Meyer et al., 2012) introduce further variables sites on transcripts (Dominissini et al., 2012; Meyer et al., 2012; Schwartz et al., 2013), which usually are not represented in full-length cDNA sequences. Over 100 different types of RNA modifications have been identified to date, and these have been shown to be involved in nearly every aspect of the mRNA life cycle (Roundtree et al., 2017). For many of these alternative site, functions are known, while for others function remains elusive.

This abundance of alternative sites and events raises a number of key questions, for many (but not all) of which, sequencing of full-length isoforms is giving and is expected to yield significant insights. 1) Which combinations of the previously mentioned variable sites are actually being generated as RNA isoforms? In theory, all these alternative sites can specify an exponential number of distinct RNA molecules by exploiting distinct combinations of the previously discussed sites; however, recent data suggest that for many (but certainly not all) genes, this is not the case. 2) Can we find the precise cell types that generate each isoform? As we will see later, single-cell approaches are beginning to offer a window into this field. 3) What is the relative timing of multiple alternative processing events within a gene? 4) With multiple long-read sequencing approaches now available, we must ask to which extent these may give different answers. This review will focus on the contributions made by long-read RNA sequencing to date and will also include a discussion of the technical challenges that have been overcome and that will need to be overcome in the future.

## Functionality of Alternative RNA Processing Events

We will only briefly touch on other important questions, such as “Which isoforms harbor function?”—a question whose negative is not easily assessed, given the variety of possible settings in which an event may be relevant. Here, we will limit ourselves to saying that there are many clear examples for the functionality of alternative RNA processing events. This is exemplified by the FAS receptor (Cheng et al., 1994) and the finding that for the majority of tested genes, distinct alternative isoforms differ in their protein interaction partners, once translated (Yang et al., 2016). Detailing all the functional consequences of alternative splicing is beyond the scope of this review, and this topic has been recently reviewed by others (Cieply and Carstens, 2015; Daguenet et al., 2015; Raj and Blencowe, 2015; Vuong et al., 2016; Fiszbein and Kornblihtt, 2017; Gallego-Paez et al., 2017; Mauger and Scheiffele, 2017).

## Limitations of Short-Read RNA Sequencing

High-throughput transcriptional profiling (“RNA-seq”) was pioneered in 2008, which enabled a transcriptome-wide survey of gene expression and alternative splicing in a quantitative fashion (Mortazavi et al., 2008; Nagalakshmi et al., 2008; Pan et al., 2008; Sultan et al., 2008; Wang et al., 2008; Wilhelm et al., 2008). Despite the success of RNA-seq in greatly expanding our knowledge of the mammalian transcriptome, it relies on short sequencing reads (∼100–150 bp), which must be computationally assembled into longer transcript models. This can be a notoriously difficult and error-prone task, particularly when alternative splicing generates multiple partially redundant isoforms at a given locus (Steijger et al., 2013; Tilgner et al., 2013). With saturating coverage, short-read RNA-seq can accurately measure percent spliced-in (PSI) scores for individual exons but cannot unambiguously resolve the connectivity between distant exons because they are never represented on the same sequenced fragment (Tilgner et al., 2015; Tilgner et al., 2018). With the emergence of third-generation sequencing, it is now possible to sequence full-length transcripts “in one go,” thereby obviating the challenges posed by computational assembly and delivering reliable isoform structures.

## A Brief History of Third-Generation Isoform Sequencing

**Figure 1:**
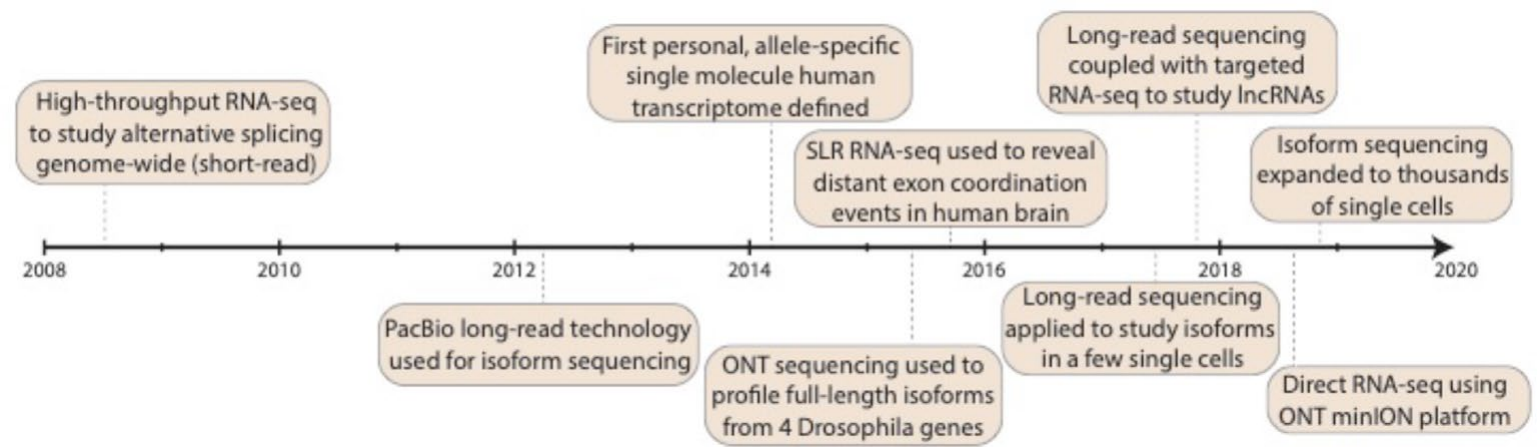
Progress in isoform sequencing. Timeline highlights some of the key milestones in the history of isoform sequencing, dating back to the advent of short-read RNA-seq back in 2008. Note that this is presented as a summary only and is not intended to be exhaustive of all work done in the field. RNA-seq: RNA sequencing; PacBio: Pacific Biosciences; SLR: synthetic long-read; lncRNA: long noncoding RNA; ONT: Oxford Nanopore Technologies.

In the second decade of the millennium, third-generation sequencing experienced and is experiencing rapid growth (**Figure 1**). This occurred with an abundance of alternative RNA processing events described (see the previous section), which set the stage for questions that could be addressed with long-read isoform sequencing. Note, this review largely ignores long-read and linked-read applications to non-transcriptome work, which have been reviewed (focusing on PacBio) in 2015 (Rhoads and Au, 2015) and considering PacBio, nanopore, and linked-read technologies in 2018 (Sedlazeck et al., 2018). The first long-read platform that truly allowed sequencing of full-length isoforms in a single read was the Pacific Biosciences (PacBio) platform (Eid et al., 2009), which started to be used for isoform descriptions in 2012. PacBio sequencing works by utilizing a DNA polymerase that is affixed at the bottom of a zero-mode waveguide (ZMW) with a single molecule of DNA as a template. As with earlier sequencing technologies, each of the four DNA nucleotides is attached to one of four different fluorescent dyes, and nucleotide incorporation is observed in real time. Many ZMWs are incorporated on a single chip, enabling massive parallelization. Koren et al. (2012) investigated the corn transcriptome using methods of error correction (see later). In 2013, Sharon et al. (2013) exploited a panel of human organs, which theoretically harbors large amounts of splice variants, to describe full-length molecules in a PCR-free fashion based on circular consensus sequences (Eid et al., 2009; Travers et al., 2010) (CCS, see later), and Au et al. (2013) described the transcriptome of human embryonic stem cells, again using error correction. In 2014, we (Tilgner et al., 2014) produced an enhanced GENCODE annotation, adding full-length isoforms from lymphoblastoid cells and from a panel of human organs to the GENCODE annotation (Harrow et al., 2006; Harrow et al., 2012). This same year (2014) also saw important work, investigating the connectivity of neurexin exons in a targeted manner (Schreiner et al., 2014; Treutlein et al., 2014). The following year (2015) saw the emergence of the first non-PacBio long-read strategies. Bolisetty et al. (2015) and Roy et al. (2015) pioneered the use of Oxford Nanopore Technologies (ONT) sequencing to study exon connectivity for a set of target genes (Bolisetty et al., 2015; Roy et al., 2015). Nanopore sequencing works by detecting changes in current that occur when a biological molecule (e.g., DNA) passes through a nanoscale pore. These changes in current (“squiggles”) are measured and then computationally converted into DNA nucleotides. We (Tilgner et al., 2015) exploited the dilution-based Moleculo approach (McCoy et al., 2014; Voskoboynik et al., 2013) for RNA sequencing to reveal nonrandomly paired alternative exon pairs genome-wide. Likewise, we developed another PacBio competitor—sparse isoform sequencing (SpISO-Seq)—which is based on linked-read sequencing (Zheng et al., 2016) and allows the description of many more millions of RNA molecules (Tilgner et al., 2018). Despite the availability of competitors, PacBio continues to be heavily used and developed for isoform sequencing (Gordon et al., 2015; Weirather et al., 2015; Shi et al., 2016; Tevz et al., 2016; Tombácz et al., 2016; Lagarde et al., 2017; Sahraeian et al., 2017; Tseng et al., 2017; Anvar et al., 2018; Balázs et al., 2018; Deveson et al., 2018; Dougherty et al., 2018; Gupta et al., 2018; Tardaguila et al., 2018; Wyman and Mortazavi, 2019). Both ONT (through its now available PromethION instrument) and PacBio (through an announced 8 million ZMW SMRT cell^1^) are poised for large throughput increases, which could dramatically alter our view of isoform biology. The year 2017 saw the first long-read strategies for a few (∼10^1^) single cells (Byrne et al., 2017; Karlsson and Linnarsson, 2017), and in 2018, we and others introduced the first applications of long-read technologies to ∼10^2^ (Volden et al., 2018) and 10^3^–10^4^ (Gupta et al., 2018) individual cells (see later).

## Characterization of lncRNAs and Their Biology Through Isoform Sequencing

Due to the relatively shallow sequencing depth provided by third-generation sequencing platforms, the majority of studies to date have focused on protein-coding genes due to their higher overall expression. Early isoform studies with third-generation sequencing studies (or “454” 400–700-bp reads) (Au et al., 2013; Sharon et al., 2013; Tilgner et al., 2013; Tilgner et al., 2014; Tilgner et al., 2015) revealed consistently novel aspects of long noncoding RNA (lncRNA) expression. Using “454” (Tilgner et al., 2013), PacBio (Sharon et al., 2013; Tilgner et al., 2014), and Moleculo (Tilgner et al., 2015) sequencing, we found that 30–40% of all long reads aligned to known GENCODE lncRNA loci were inconsistent with all annotated isoforms for the loci in question. This was dramatically higher than for long reads aligned to protein-coding loci. A simple explanation for these observations appeared to be that lncRNAs had been less comprehensively investigated than protein-coding genes. Therefore, increased novelty rates would be simply a reflection of our more limited (in comparison with protein-coding genes) knowledge of lncRNA biology. Recent research has shed new light on this observation: the Mercer and Mattick laboratories found universal alternative splicing of noncoding exons (Deveson et al., 2018), including those in lncRNAs. That is, unlike protein-coding exons, almost all noncoding exons were found to be alternatively spliced (i.e., had a PSI score < 95%). This suggests that splicing patterns in lncRNAs may not fall under the same level of constraint as those in protein-coding genes; as the requirement to maintain an ORF is not imposed on noncoding RNA, this allows the spliceosome to explore the full range of noncoding exon combinations available. This would in turn explain the much larger fraction of lncRNA long reads that are inconsistent with all annotated isoforms. In summary, lncRNA isoforms appear to exploit all exons as alternative when interrogated in bulk tissue. A key question now is whether the previous observation could change profoundly when considering highly specific cell types or cell populations. In other words, there are two scenarios that could lead to the observation of universal alternative splicing in noncoding exons: 1) Within specific cell populations, splicing of these noncoding exons may be constitutive but different between populations; 2) splicing of these noncoding exons could also be alternative within all cell populations.

Simultaneous with the previous results, the Wong laboratory’s hybrid sequencing approach (Au et al., 2013) (i.e., combining both short- and long-read sequencing technologies) revealed 216 novel gene loci, which were unknown to GENCODE (Harrow et al., 2006; Harrow et al., 2012), RefSeq (O’Leary et al., 2016), and the UCSC (Karolchik et al., 2014) annotation. A subset of these were lncRNAs preferentially expressed in pluripotent cell lines (Au et al., 2013), and a further subset of three of such lncRNAs was later shown to play a role in preimplantation embryo development (Durruthy-Durruthy et al., 2015). Earlier work had established a number of characteristic features of intergenic lncRNAs, including (but not limited to) a lower number of exons and a shorter transcript length compared with those of protein-coding transcripts (Derrien et al., 2012). Targeted RNA capture (Clark et al., 2015) in conjunction with PacBio long-read sequencing, however, increased the estimates of average lncRNA transcript length and exon number substantially, although the estimates for protein-coding genes were not entirely matched (Lagarde et al., 2017). This work by the GENCODE consortium vastly improved lncRNA annotations, with the number of lncRNA genes and transcripts now easily outnumbering their protein-coding counterparts. Another way of posing the previous key question for the future is whether specific lncRNA isoforms are characteristic of precise cell populations.

## Combination Patterns and Timing of Multiple RNA Processing Events

**Figure 2:**
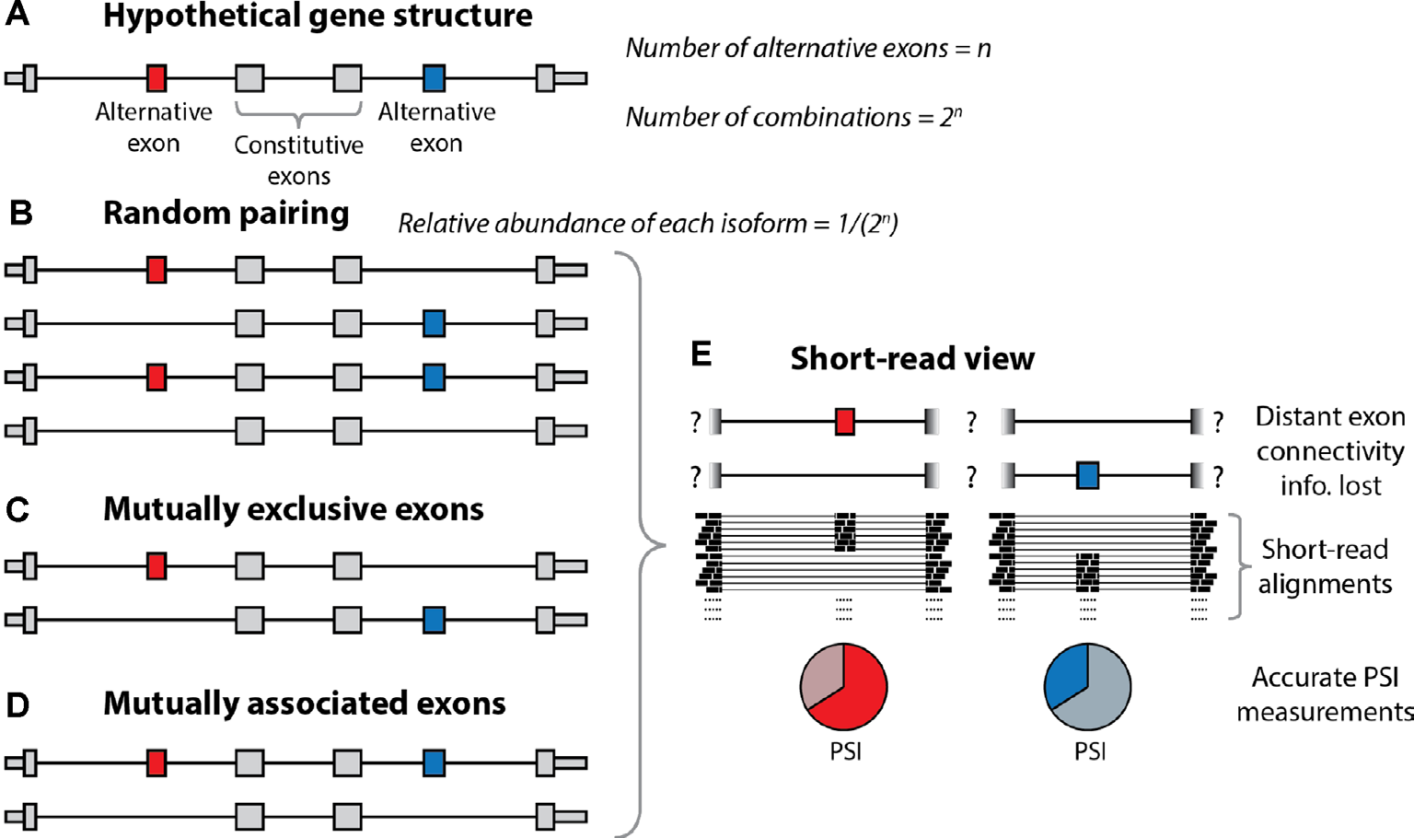
Resolution of alternative splicing events with long-read sequencing. **(A)** Schematic illustration of the structure of a hypothetical gene undergoing alternative splicing. The gene contains two alternatively spliced exons (red and blue) separated by constitutive exons (gray). In theory, if we let *‘n’* equal the number of alternative exons, then there are *2* *^n^* different combinations of these exons. **(B)** Under random pairing, we would expect to see all of these *2* *^n^* combinations, each at a relative abundance of *1/2* *^n^*. In this case, short-read RNA-seq would be sufficient, as it can accurately quantify percent spliced-in (PSI) scores for individual exons. **(C**, **D)** However, coordinated exon pairing can result in a situation whereby the alternative exons are mutually exclusive **(C)** or mutually associated **(D)**. **(E)** With short-read RNA-seq, these three scenarios are indistinguishable, as the information regarding the connectivity of the alternative exons is lost. Conversely, with long-read sequencing, it is trivial to determine which scenario is present.

As noted previously, a single gene can harbor multiple distinct alternative exons and other alternative processing events. Let us consider a hypothetical gene with *n* alternative sites with only two options each, which (for simplicity of the argument) are all used in 50% of the molecules (**Figure 2**). At one extreme, of the spectrum of possible combinations, these *n* exons (or more general sites) could produce 2*^n^* combinations at relative abundances of 1/(2*^n^*) through exhaustive random pairing. Under random pairing, short-read sequencing would be the method of choice because it would yield a usage probability for each variable site at the lowest cost. The frequency of each complete isoform could then be determined by multiplication over the associated probabilities. At the other end of the spectrum, perfectly coordinated exon pairing could result in just two isoforms (e.g., the isoform including all *n* exons and the one skipping all *n* exons). In this setting, short-read probabilities of variable sites would be uninformative for complete isoforms. The interest of combinations has been noticed and investigated for a long time (MacLeod et al., 1985; Helfman et al., 1986; Cramer et al., 1997; Fededa et al., 2005), revealing the combination patterns of such alternative processing RNA sites. Thus, Helfman et al. observed nonrandom pairing of an internal exon and a 3’ exon (Helfman et al., 1986), Cramer et al. (1997) showed the dependence of inclusion of an internal exon on promoter structure, and Fededa et al. (2005) showed the dependent splicing outcome of two alternative exons in the *fibronectin* gene. Most interestingly, these authors showed that inclusion levels of the upstream alternative exon conditioned inclusion levels of the downstream one but not in the opposite way, which revealed a gene polarity mechanism probably due to changes in RNA polymerase II elongation rates. At a genome-wide level, Fagnani et al. (2007) revealed correlated exon inclusion using short-reads, but this approach did not allow them to distinguish between two models: A) One type of molecule would include exon 1 and another would include exon 2, with upregulation of both types (at the expense of molecules including both or none of the exons) leading to the observation of correlation. B) Preferential expression of molecules employing either both or none of the exons. In 2014, the neurexin genes were investigated using PacBio by the Sudhof and Scheiffele laboratories (Schreiner et al., 2014; Treutlein et al., 2014), with nonadjacent exons being mostly randomly paired. In 2015, we employed deep long-read sequencing of ∼5 million ∼2-kb reads (Tilgner et al., 2015) to reveal >100 human genes with coordinated pairs of alternative exons. Consistent with the previously discussed work, neurexins could not be shown to harbor any nonrandom pairing of nonadjacent exons. That same year, the Graveley and Moore labs employed ONT sequencing to establish the connectivity of alternative exons in a mouse gene and the *Drosophila Dscam* gene (Roy et al., 2015) as well as four *Drosophila* genes (Bolisetty et al., 2015). Finally, we recently estimated that 40% of genes with multiple distant alternative splicing events show coordination of these events (Tilgner et al., 2018), and Anvar et al. (2018) revealed thousands of coordination events between exons (including adjacent exon pairs), TSS, and polyA-sites. Interestingly, coordination of distant splicing events in bulk tissue occurs in the presence of different isoform expression between cell types more frequently than coordination of adjacent exons (Gupta et al., 2018). In summary, these combination patterns generally warrant the use of long-read strategies for isoform descriptions.

The combination patterns of *n* alternative binary processing events can result in 2*^n^* combinations. However, the relative order of *n* events could in principle be carried out in *n!* distinct orders, which defines an even greater search space, than the previously mentioned 2*^n^* combinations. As a general background, it is widely appreciated that splicing occurs very frequently co-transcriptionally, that is, while the RNA molecule is still in proximity to the chromatin template (Beyer and Osheim, 1988; Ameur et al., 2011; Khodor et al., 2011; Tilgner et al., 2012; Schor et al., 2013). An important finding in this realm, involving third-generation sequencing, was recently revealed by Carillo Oesterreich and colleagues, who employed PacBio sequencing to track the splicing status of introns in yeast. These authors revealed that once RNA polymerase has transcribed 45 nt of downstream DNA, half of the preceding introns have undergone splicing in yeast (Carrillo Oesterreich et al., 2016), implying very fast intron removal. The same laboratory more recently aimed at investigating the order of intron removal in the fission yeast *Schizosaccharomyces pombe*, revealing most multi-intron transcripts to be fully spliced or fully unspliced (Herzel et al., 2018).

## Direct RNA Sequencing

Until recently, high-throughput RNA-seq assays have relied on an initial step in which the RNA is first converted to cDNA before sequencing. Thus, these methods detect the products of a synthesis reaction rather than directly reading the RNA molecule itself. Crucially, any RNA modifications are lost in the process of cDNA conversion. While the first direct RNA sequencing method was published almost a decade ago—the Helicos platform (Ozsolak et al., 2009)—this method relied on short sequence reads. Long-read direct RNA sequencing provides a framework in which TSSs, splice sites, polyA-sites, RNA-editing, as well as a number of RNA modifications, whose positions are lost during reverse transcription, can theoretically be interrogated simultaneously on single molecules. This can advance the identification of single sites but, above all, can also reveal the combination patterns of all these different alterations. Recently, ONT has provided proof of concept of direct RNA sequencing in yeast (Garalde et al., 2018), showing that the MinION platform can detect all the previously mentioned variables that define the sequence of an RNA molecule. The Nanopore WGS Consortium (Workman et al., 2018) has recently extended this technique to directly sequence a human polyA transcriptome, impressively generating ∼10 million aligned sequence reads that were filtered into ∼78,000 high-confidence isoforms (the majority of which contained novel splice junctions missing from GENCODE). However, for the moment, it appears difficult to define all variable sites accurately, based on a single read only.

## Isoform Sequencing From Single Cells

Short-read single-cell splicing studies had revealed the existence of bimodality for percent spliced-in (PSI) distributions across individuals cells (Shalek et al., 2013). That is, individual cells of similar type could differ drastically in their inclusion of a specific exon. More recent work (Song et al., 2017) showed that 20% of alternative exons show this phenotype of bimodality. The advent of long-read third-generation sequencing made it only natural to wonder if full-length isoforms could be profiled from individual cells. Thus, Karlsson and Linnarsson (2017) employed PacBio sequencing to monitor isoforms in six individual mouse brain cells (one vascular, one leptomeningeal, and four oligodendrocyte type cells in different maturation stages) and revealed strong isoform diversity within single cells. The Vollmers lab (Byrne et al., 2017) used and benchmarked the ONT system on seven individual B-cells and found widespread usage of novel TSSs and transcription end sites (TESs), as well as 100–1,000 alternative splicing events.

In 2018, the same lab extended the single-cell long-read view to 96 cells (Volden et al., 2018), also describing a CCS-like method for nanopore sequencing (see previous discussion). Still in 2018, our laboratory described singe-cell isoform RNA sequencing for 5,000–10,000 cells (Gupta et al., 2018), which produces complete cDNAs tagged for their cell of origin (here by using 10x Genomics) (Zheng et al., 2017), and PacBio or ONT to produce full-length isoforms. By identifying barcodes in each long read, one can assign each read to its cell of origin. The advantage of this last approach is that the number of cells (>5,000) allows clustering of cells into cell types and, therefore, an isoform description of all (sufficiently abundant) cell types in a bulk sample. This technology enables a wealth of applications: First, it allows the tracing of the effect of single nucleotide polymorphisms (SNPs) (or germline mutations) into distinct cell types. Interestingly, such sequence alterations are, in principle, present in every single cell and cell type and may affect genes that are expressed across multiple cell types. By sequencing isoforms of thousands of single cells, we may be able to understand to which extent the action of such SNPs differs across cell types or single cells. Second, in case–control settings of diseases, we may be able to trace the consequences of disease-causing genome alterations or environmental factors into specific cell types—which may pave the way for devising strategies that “correct” isoform regulation in a cell-type specific way.

## Parameters of Isoform Sequencing

The advantages and disadvantages of different long-read sequencing strategies can be summarized using several criteria (summarized in **Supplementary Table 1**). In this review, we will not attempt to mathematically define these but rather to explain the intuition behind them. These measurements include “completeness of reads,” “correctness of sequence,” “bias of representation,” “sequencing depth,” and the “minimal input amount.”

1. *Completeness of reads:* Completeness of reads describes the extent to which a long read represents the entire underlying RNA molecule. An interesting twist to this question is that a long read may represent a complete RNA molecule (which was turned into cDNA) but not a complete transcript, as the RNA molecule may have suffered damage in the cell or during the experiment. Whether a read is complete at its 3’ end is, in theory, relatively easily assessed by considering its polyA-tail. Of note, a cDNA molecule that is generated through reverse transcription with a polydT primer must contain a polyT (or polyA depending on its orientation) region at its end. This is the case even if the polydT primer annealed to a non-perfect genomic A-rich region because the sequence in the cDNA is determined by the primer, not by the transcript’s region that the primer bound to. Given these observations, it was a surprise that we initially, using a hidden Markov model, only found 67% of PacBio CCSs to contain a polyA-tail (Sharon et al., 2013). Broadly consistently, we recently found 61.4% of single-cell long reads to contain a polyA-tail, and Lagarde et al. (2017) report 73% (human) and 64% (mouse) of all reads of insert to yield an identifiable polyadenylation site. Given the previously discussed considerations, it is likely that the missing polyA-tails are lost during CCS generation or possibly earlier in the experiment. A measure of completeness that applies to both 5’ and 3’ end of reads can be obtained through the comparison with annotated transcripts. This measure is, however, intrinsically subject to the completeness and correctness of the employed annotation (Sharon et al., 2013; Tilgner et al., 2014; Tilgner et al., 2015; Uszczynska-Ratajczak et al., 2018).

2. *Correctness of sequence:* PacBio and ONT raw reads have much higher per base error rates than Illumina sequencing. Linked-read-based methods exploit the repetitive sequencing of individual cDNA molecules to reach quality comparable (and superior) with Illumina short reads (Tilgner et al., 2015; Tilgner et al., 2018). However, there is far less support software available. For PacBio and ONT, the error rates in raw reads have ranged from 10 to 20% but are subject to change in the future. There are currently three approaches to limit the consequences of these error rates. A) The first relies on building CCSs of lower error rates from multiple low-quality read outs of the same molecule. This has been pioneered by PacBio (Eid et al., 2009; Travers et al., 2010) and has been widely used ever since. The advantage of this approach is that all the information in an individual CCS originates from one original RNA molecule, with the disadvantage being that reads shorter than the molecule of interest cannot generate such CCS. While ignoring such reads may introduce a bias against long molecules, the ever-increasing read length of PacBio is likely to increase CCS numbers. For PacBio, the CCS approach has gone through rounds of optimization. For ONT, a recent report (Li et al., 2016) engineered a CCS-like strategy: circularization of molecules and rolling-circle amplification generated long molecules, which repeatedly contain the molecule of interest. Sequencing of this repeat allowed the construction of consensus reads, similar to PacBio CCS. Volden et al. (2018) applied this approach to mRNA, obtaining an accuracy of 94%. This is considerably higher than raw ONT accuracy but still lags behind PacBio CCS accuracy. Possibly, algorithmic improvements to the method could raise the 94% accuracy, although the nonrandom nature of ONT errors could limit such improvements. B) The second approach employs higher-quality short Illumina reads to correct errors in higher-quality long reads. This method was first used in 2012 (Au et al., 2012; Koren et al., 2012) and is also employed in recent software, including LoRDEC (Salmela and Rivals, 2014) and *proovread* (Hackl et al., 2014). This approach has the advantage of rescuing many long reads that cannot form consensus and that would otherwise be lost, with the disadvantage being that the resulting corrected long read is a hybrid of multiple distinct molecules that may not harbor identical sequence. A relatively recent publication has compared the effects of error correction on PacBio and ONT reads (Weirather et al., 2017), although the employed data predate both the PacBio Sequel and the ONT PromethION. C) Last but not least, consensus and error correction can also be achieved from multiple long reads after grouping similar long reads. This simplifies experimental procedures, as only one sequencing experiment has to be performed. However, if systematic biases are present in the original reads, these could persist in the final consensus. Relevant tools include Tofu (Gordon et al., 2015), TAPIS (Abdel-Ghany et al., 2016), and CARNAC-LR (Marchet et al., 2019).

3. *Bias of representation:* Different cDNA molecules can differ in a variety of characteristics, including length, sequence (often summarized as GC) content, structure, to name only a few. Looking at the bias of representation fundamentally asks whether the molecules that are presented to the machine differ significantly in any of the previously discussed characteristics from those that are reported as long reads. On the PacBio machine, there is little to no bias of coverage in GC-rich region (Ferrarini et al., 2013); however, there is a bias for shorter molecules. This length bias has been counteracted by sequencing distinct size selections (Chin et al., 2013), which ensures that larger molecules are not lost due to preferential sequencing of shorter molecules. The ONT system was recently tested (Oikonomopoulos et al., 2016) on the External RNA Controls Consortium (ERCC) synthetic spike-ins (Baker et al., 2005), observing no length or GC bias (see later discussion) and then applied to human HEK-293 cells. However, it is worth noting that the longest ERCC spike-in transcript is only ∼2 kb in length. ONT sequencing was recently benchmarked using “sequin” spike-ins (Hardwick et al., 2016; Hardwick et al., 2019), which include 15 multi-exonic transcripts in the 2.5–7-kb range.

4. *Sequencing depth:* Sequencing depth has been the Achilles’ heel of isoform sequencing for a long time. Our initial PacBio isoform sequencing paper yielded ∼500,000 (Sharon et al., 2013) CCS reads, and we then increased this to ∼2 million a year later (Tilgner et al., 2014). These limitations (along with required input amount) were our primary motivation to explore dilution-based methods (Tilgner et al., 2015; Tilgner et al., 2018), which yielded 5 and 25 million long reads, respectively. It now seems that a breakthrough has been achieved with the PromethION from ONT, which at the time of writing appears to yield 20–50 million long reads, although currently at lower quality. Likewise, PacBio has announced an 8-million ZMW SMRT cell.

5. *Minimal input amount:* Both PacBio and now ONT require large amounts of input material. This requires either starting with large amounts of material or extensive PCR, the latter of which of course can decrease library complexity and introduce quantitative bias. Rolling-circle PCR, however, such as used by Volden et al. (2018), is an attractive work-around, as it amplifies molecules, while ensuring that all copies of the original cDNA molecule are sequenced in one single read. Therefore, no PCR duplicates are created unless standard PCR is used before or after. Dilution-based isoform sequencing methods (Tilgner et al., 2015; Tilgner et al., 2018) start with 100 pg to 1 ng. While there is PCR involved, this PCR occurs in barcoded wells or droplets, and all reads originating from one molecule can be collapsed back onto the original molecule.

As for deciding which sequencing platform to use, it largely depends on the specific goals and priorities of the study. For example, if high splice site accuracy is needed and money is no object, then PacBio arguably remains the best option in most cases. While ONT sequencing has a relatively high error rate, this may be tolerable in cases where perfect splice site accuracy is not required. If the goal is to detect RNA modifications or perform direct RNA sequencing, then ONT is the method of choice. Likewise, if portability is essential—e.g., for use in the field—then, ONT’s MinION device is recommended.

## Mapping of Long Reads

The long, noisy sequencing reads produced by third-generation technologies have posed new bioinformatic challenges for accurate spliced read alignment (Križanović et al., 2018). This has necessitated the development of specialized long-read alignment tools, the most popular of which currently include GMAP (Wu and Watanabe, 2005), STAR (Dobin et al., 2013), BLASR (Chaisson and Tesler, 2012), Minimap2 (Li, 2018), and Magic-BLAST (Boratyn et al., 2018). All of these aligners are splice-aware with the exception of BLASR, which was designed for alignment of genome sequencing data and, thus, is not recommended for RNA sequencing. The performance of GMAP and STAR was comprehensively benchmarked for PacBio long reads in the Association of Biomolecular Research Facilities (ABRF) next-generation sequencing study (Li et al., 2014). This study, however, predates the advent of nanopore sequencing, the introduction of the PacBio Sequel instrument, as well as the introduction of the Minimap2 and Magic-BLAST software. A detailed study of the performance of these mappers, especially with respect to accuracy on PacBio and ONT reads, would therefore be of high interest. The higher error rates of long-read sequencing platforms can also confound the precise determination of splice junctions. This has led to the emergence of several tools designed to cluster, polish, and collapse long reads into high-confidence isoforms, including Mandalorion (Byrne et al., 2017), Carnac-LR (Marchet et al., 2019), Pinfish^2^, and FLAIR (Tang et al., 2018). Some of these tools can optionally be run in conjunction with short-read RNA-seq data to help increase accuracy of splice junction detection and quantification.

## Concluding remarks

RNA molecules can be multiple kilobases long and even up to 100 kilobases if premature molecules are considered. Yet, for the first decade of the new millennium, close to all transcriptome-wide approaches worked on RNA or cDNA fragments. From 2010 on, a revolution started that allowed the consideration of full-length RNA molecules, and at the time of writing, the resulting technologies are on the verge of going mainstream. The ultimate goal is to unambiguously decipher the sequence, structure, and abundance of each RNA molecule produced by a given cell, including its TSS, splicing structure, RNA modifications, and poly-A tail. Moving forward, the main technical challenges that will need to be overcome are the relatively high error rates, low throughput, and large input material requirements (compared with short-read RNA-seq). The continued development of novel bioinformatic approaches designed specifically for long, noisy reads can be expected to lead to further increases in performance. It seems that in the near future, a lot of biological reasoning could be performed with the isoform as a unit, rather than with single exons, splice sites, RNA edits, and modifications. Eventually, this development will hopefully further the large body of knowledge on the interactions between the respective machineries and allow us to appreciate all variables on individual RNA molecules at once.

## Author Contributions

All authors listed have made substantial, direct, and intellectual contribution to the work and approved it for publication.

## Funding

HT is a Leon Levy Research Fellow in Neuroscience and is furthermore grateful for a generous gift by Anita Garoppolo. SH acknowledges an Australian National Health and Medical Research Council (NHMRC) Early Career Fellowship (APP1156531). PF and AF acknowledge support from the National Human Genome Research Institute (U41HG007234), the Wellcome Trust (WT108749/Z/15/Z), and the European Molecular Biology Laboratory. PF is a member of the Scientific Advisory Boards of Fabric Genomics, Inc., and Eagle Genomics, Ltd.

## Conflict of Interest Statement

PF is a member of the Scientific Advisory Boards of Fabric Genomics, Inc., and Eagle Genomics, Ltd. The remaining authors declare that the research was conducted in the absence of any commercial or financial relationships that could be construed as a potential conflict of interest.

## Footnotes

1 https://www.pacb.com/press_releases/pacific-biosciences-launches-new-sequel-ii-system-featuring-8-times-the-dna-sequencing-data-output/.

2 https://github.com/nanoporetech/pinfish.
