# Supplementary material for: Getting the Entire Message: Progress in Isoform Sequencing
Source: Front Genet. 2019 Aug 16;10:709. doi: 10.3389/fgene.2019.00709 (PMC6706457; doi:10.3389/fgene.2019.00709)
Supplement: Supplementary file 1 [file Table_1.docx]

**Supplementary Table 1 | Outline of the main third-generation isoform sequencing platforms.**

Table shows the main third-generation isoform sequencing platforms that have been developed to date, including performance metrics and advantages/disadvantages.

| **Platform** | **Read**  **length** | **Error rate** | **Sequencing**  **depth** | **Input cDNA** | **Advantages** | **Disadvantages** |
| --- | --- | --- | --- | --- | --- | --- |
| PacBio | ~0.5 - 10 kb | - ~10% (raw) - ~1% (CCS) | - 200K - 400K CCS/1M ZMW SMRT cell (Sequel) - 8M ZMW SMRT cell (to be released soon) | ~10^-6^ g | - No GC bias. - High accuracy (with CCS reads). | Biased towards shorter molecules (RS and RS2 machines). |
| SLR  (Moleculo) | ~0.5 - 8 kb | <1% | 500K/Illumina lane | ~10^-9^ g | - Low error rate. - Number of lanes can be chosen. | Not truly ‘single molecule’, as molecules are barcoded before short-read sequencing. |
| SLR  (10x) | ~0.5 - 8 kb | <1% | 25M reads (sequenced on 7 Illumina lanes) | ~10^-9^ g | Greater read depth. | - As above. - Requires large number of molecules and lanes. |
| ONT | ~0.5 - 10 kb (but theoretically unlimited) | ~10 - 15% | - 0.5 - 2M reads (MinION) - 50M reads (PromethION) | ~10^-6^ g | - No length or GC bias. - Enables detection of RNA modifications. - Can be used for direct RNA sequencing. - Highly portable (MinION). | High error rate (particularly indels). |

**Abbreviations:**

PacBio: Pacific Biosciences; CCS: Circular consensus sequencing; ZMW: Zero-mode waveguide; SLR: Synthetic long-read; ONT: Oxford Nanopore Technologies.
